# 2-Carb­oxy-1-phenyl­ethanaminium nitrate

**DOI:** 10.1107/S1600536809042792

**Published:** 2009-10-28

**Authors:** Wen-Xian Liang, Xiao-Wei Chu, Zhi-Rong Qu

**Affiliations:** a Ordered Matter Science Research Center, College of Chemistry and Chemical, Engineering, Southeast University, Nanjing 210096, People’s Republic of China

## Abstract

In the title salt, C_9_H_12_NO_2_^+^·NO_3_^−^, the cation and anion are linked by a bifurcated N—H⋯(O,O) hydrogen bond. The crystal packing is stabilized by inter­molecular N—H⋯O, O—H⋯O and C—H⋯O hydrogen bonds, which connect neighbouring cations and anions, resulting in a two-dimensional network.

## Related literature

For details of the preparation of β-amino acids, see: Cohen *et al.* (2002 ▶); Qu *et al.* (2004 ▶).
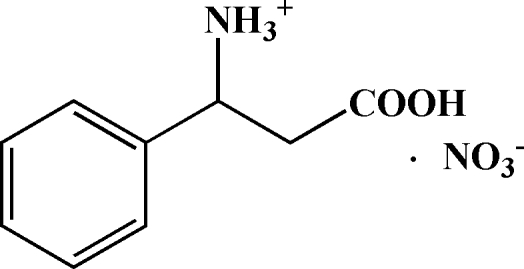


## Experimental

### 

#### Crystal data

C_9_H_12_NO_2_^+^·NO_3_^−^
*M* *_r_* = 228.21
Monoclinic, 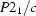

*a* = 6.2017 (12) Å
*b* = 10.313 (2) Å
*c* = 18.077 (4) Å
β = 105.36 (3)°
*V* = 1114.9 (4) Å^3^
*Z* = 4
Mo *K*α radiation
μ = 0.11 mm^−1^
*T* = 293 K
0.50 × 0.30 × 0.15 mm

#### Data collection

Rigaku SCXmini diffractometer
Absorption correction: multi-scan (*CrystalClear*; Rigaku, 2005 ▶) *T*_min_ = 0.960, *T*_max_ = 0.982
11050 measured reflections
2549 independent reflections
1652 reflections with *I* > 2σ(*I*)
*R*_int_ = 0.061

#### Refinement

*R*[*F* ^2^ > 2σ(*F* ^2^)] = 0.080
*wR*(*F* ^2^) = 0.231
*S* = 1.11
2549 reflections
149 parameters
1 restraint
H-atom parameters constrained
Δρ_max_ = 0.59 e Å^−3^
Δρ_min_ = −0.23 e Å^−3^

### 

Data collection: *CrystalClear* (Rigaku 2005 ▶); cell refinement: *CrystalClear*; data reduction: *CrystalClear*; program(s) used to solve structure: *SHELXS97* (Sheldrick, 2008 ▶); program(s) used to refine structure: *SHELXL97* (Sheldrick, 2008 ▶); molecular graphics: *SHELXTL* (Sheldrick, 2008 ▶); software used to prepare material for publication: *PRPKAPPA* (Ferguson, 1999 ▶).

## Supplementary Material

Crystal structure: contains datablocks I, New_Global_Publ_Block. DOI: 10.1107/S1600536809042792/sj2656sup1.cif

Structure factors: contains datablocks I. DOI: 10.1107/S1600536809042792/sj2656Isup2.hkl

Additional supplementary materials:  crystallographic information; 3D view; checkCIF report

## Figures and Tables

**Table 1:** Hydrogen-bond geometry (Å, °)

*D*—H⋯*A*	*D*—H	H⋯*A*	*D*⋯*A*	*D*—H⋯*A*
C8—H8⋯O2^i^	0.93	2.56	3.414 (5)	152
C2—H2*A*⋯O4^ii^	0.97	2.46	3.256 (5)	140
O2—H2⋯O5^iii^	0.82	2.01	2.743 (4)	148
N1—H1*C*⋯O5^ii^	0.88	2.13	2.979 (4)	160
N1—H1*C*⋯O4^ii^	0.88	2.41	3.129 (4)	139
N1—H1*B*⋯O1^iv^	0.88	1.97	2.830 (4)	166
N1—H1*A*⋯O4	0.88	2.39	3.101 (4)	138
N1—H1*A*⋯O3	0.88	2.07	2.933 (4)	165

Symmetry codes: (i) 
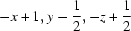
; (ii) 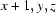; (iii) 
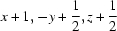
; (iv) 
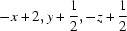
.

**Fig. 1.**
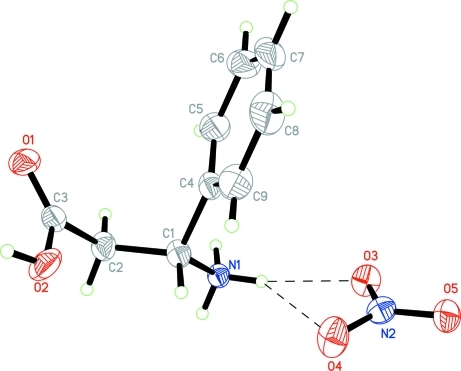
The asymmetric unit of the title compound, with displacement ellipsoids drawn at the 30% probability level. Intramolecular hydrogen bonds are drawn as a dashed lines.

**Fig. 2.**
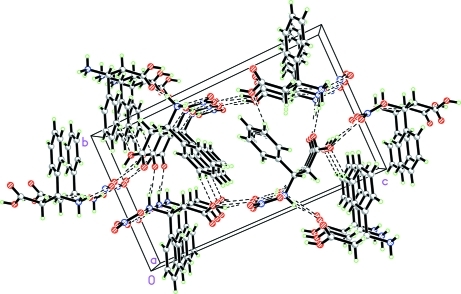
Packing diagram of the title compound, showing the structure along the b axis. Hydrogen bonds are drawn as dashed lines.

## supplementary crystallographic information

### Comment

β-Amino acids are important molecules due to their pharmacological properties. Recently, there has been an increased interest in the enantiomeric preparation of β-amino acids as precursors for the synthesis of novel biologically active compounds (Cohen *et al.*, 2002; Qu *et al.* 2004).

The title compound C_9_H_12_NO_2_^+^.NO_3_^-^ exists as two independent ions linked by bifurcated N—H···O hydrogen bonds (Fig. 1).  The crystal structure is stabilized by intermolecular N—H···O, O—H···O and C—H ···O hydrogen bonds (Table 1) which connect neighbouring cations and anions, resulting in a two-dimensional network (Fig. 2).

### Experimental

Benzaldehyde (1.59 g, 15 mmol), malonic acid (2.5 g, 24 mmol) and ammonium acetate (3.0 g, 39 mmol) were added in a flask under nitrogen and refluxed for 24 h yielding a white precipitate. After cooling to room temperature, the solution was filtered to yield 3-3mino-3-phenylpropionic acid. This was dissolved in ethanol and nitric acid. After slowly evaporating over a period of 5 d, colorless prism-like crystals of the title compound, suitable for X-ray diffraction experiments were isolated.

### Refinement

Positional parameters of all the H atoms were calculated geometrically and were allowed to ride on the C, N atoms to which they are bonded, with C—H = 0.93 to 1.00 Å, *U*_iso_(H) = 1.2 *U*_eq_(C), N—H = 0.88 Å, *U*_iso_(H)= 1.5 *U*_eq_(N).

### Crystal data

**Table:**

C_9_H_12_NO_2_^+^·NO_3_^−^	*F*(000) = 480
*M**_r_* = 228.21	*D*_x_ = 1.360 Mg m^−^^3^
Monoclinic, *P*2_1_/*c*	Mo *K*α radiation, λ = 0.71073 Å
Hall symbol: -P 2ybc	Cell parameters from 1797 reflections
*a* = 6.2017 (12) Å	θ = 3.1–27.5°
*b* = 10.313 (2) Å	µ = 0.11 mm^−^^1^
*c* = 18.077 (4) Å	*T* = 293 K
β = 105.36 (3)°	Prism, colorless
*V* = 1114.9 (4) Å^3^	0.50 × 0.30 × 0.15 mm
*Z* = 4	

### Data collection

**Table:**

Rigaku SCXmini diffractometer	2549 independent reflections
Radiation source: fine-focus sealed tube	1652 reflections with *I* > 2σ(*I*)
graphite	*R*_int_ = 0.061
Detector resolution: 13.6612 pixels mm^-1^	θ_max_ = 27.5°, θ_min_ = 3.1°
CCD_Profile_fitting scans	*h* = −8→8
Absorption correction: multi-scan (*CrystalClear*; Rigaku, 2005)	*k* = −13→13
*T*_min_ = 0.960, *T*_max_ = 0.982	*l* = −23→23
11050 measured reflections	

### Refinement

**Table:**

Refinement on *F*^2^	Primary atom site location: structure-invariant direct methods
Least-squares matrix: full	Secondary atom site location: difference Fourier map
*R*[*F*^2^ > 2σ(*F*^2^)] = 0.080	Hydrogen site location: inferred from neighbouring sites
*wR*(*F*^2^) = 0.231	H-atom parameters constrained
*S* = 1.11	*w* = 1/[σ^2^(*F*_o_^2^) + (0.0829*P*)^2^ + 1.0943*P*] where *P* = (*F*_o_^2^ + 2*F*_c_^2^)/3
2549 reflections	(Δ/σ)_max_ < 0.001
149 parameters	Δρ_max_ = 0.59 e Å^−^^3^
1 restraint	Δρ_min_ = −0.23 e Å^−^^3^

### Special details

**Table:**

Geometry. All e.s.d.'s (except the e.s.d. in the dihedral angle between two l.s. planes) are estimated using the full covariance matrix. The cell e.s.d.'s are taken into account individually in the estimation of e.s.d.'s in distances, angles and torsion angles; correlations between e.s.d.'s in cell parameters are only used when they are defined by crystal symmetry. An approximate (isotropic) treatment of cell e.s.d.'s is used for estimating e.s.d.'s involving l.s. planes.
Refinement. Refinement of *F*^2^ against ALL reflections. The weighted *R*-factor *wR* and goodness of fit *S* are based on *F*^2^, conventional *R*-factors *R* are based on *F*, with *F* set to zero for negative *F*^2^. The threshold expression of *F*^2^ > σ(*F*^2^) is used only for calculating *R*-factors(gt) *etc*. and is not relevant to the choice of reflections for refinement. *R*-factors based on *F*^2^ are statistically about twice as large as those based on *F*, and *R*- factors based on ALL data will be even larger.

### Fractional atomic coordinates and isotropic or equivalent isotropic displacement parameters (Å^2^)

**Table:**

	*x*	*y*	*z*	*U*_iso_*/*U*_eq_	
O1	1.1549 (5)	0.1241 (2)	0.30410 (14)	0.0613 (8)	
O2	0.8967 (5)	0.2333 (3)	0.34151 (16)	0.0631 (8)	
H2	0.9252	0.1813	0.3771	0.095*	
N1	0.7398 (5)	0.3900 (2)	0.11238 (14)	0.0382 (6)	
H1A	0.6020	0.3868	0.0823	0.066 (12)*	
H1B	0.7688	0.4697	0.1305	0.050 (10)*	
H1C	0.8352	0.3690	0.0856	0.050 (11)*	
C3	1.0254 (6)	0.2102 (3)	0.29593 (19)	0.0466 (8)	
C4	0.6953 (6)	0.1620 (3)	0.14746 (19)	0.0442 (8)	
C1	0.7597 (6)	0.2980 (3)	0.17750 (18)	0.0434 (8)	
H1	0.6540	0.3255	0.2063	0.052*	
C2	0.9930 (6)	0.3062 (3)	0.23070 (19)	0.0505 (9)	
H2A	1.1015	0.2890	0.2019	0.061*	
H2B	1.0190	0.3933	0.2514	0.061*	
C7	0.5759 (11)	−0.0891 (4)	0.0971 (3)	0.0857 (17)	
H7	0.5361	−0.1733	0.0805	0.103*	
C8	0.4647 (9)	−0.0261 (5)	0.1410 (4)	0.0903 (18)	
H8	0.3468	−0.0675	0.1541	0.108*	
C5	0.8088 (8)	0.0973 (4)	0.1026 (2)	0.0620 (11)	
H5	0.9274	0.1374	0.0892	0.074*	
C9	0.5224 (7)	0.1002 (4)	0.1675 (3)	0.0649 (11)	
H9	0.4448	0.1417	0.1982	0.078*	
C6	0.7458 (10)	−0.0285 (5)	0.0774 (3)	0.0817 (16)	
H6	0.8219	−0.0711	0.0466	0.098*	
O3	0.3218 (4)	0.3769 (3)	−0.01018 (15)	0.0562 (7)	
O4	0.2293 (5)	0.3826 (3)	0.09586 (15)	0.0706 (9)	
O5	−0.0245 (4)	0.3499 (3)	−0.00958 (16)	0.0597 (7)	
N2	0.1772 (5)	0.3697 (3)	0.02545 (17)	0.0457 (7)	

### Atomic displacement parameters (Å^2^)

**Table:**

	*U*^11^	*U*^22^	*U*^33^	*U*^12^	*U*^13^	*U*^23^
O1	0.094 (2)	0.0400 (14)	0.0476 (15)	0.0168 (14)	0.0138 (14)	0.0082 (11)
O2	0.0649 (18)	0.0546 (16)	0.0658 (18)	0.0237 (13)	0.0100 (14)	0.0187 (13)
N1	0.0436 (15)	0.0318 (14)	0.0352 (14)	−0.0020 (11)	0.0035 (12)	0.0044 (11)
C3	0.060 (2)	0.0397 (18)	0.0345 (17)	0.0015 (16)	0.0034 (15)	−0.0028 (14)
C4	0.053 (2)	0.0337 (16)	0.0381 (17)	−0.0049 (14)	−0.0023 (15)	0.0075 (14)
C1	0.0523 (19)	0.0366 (17)	0.0377 (17)	−0.0064 (14)	0.0055 (14)	0.0062 (13)
C2	0.059 (2)	0.0430 (19)	0.043 (2)	−0.0033 (16)	0.0017 (17)	0.0061 (15)
C7	0.110 (4)	0.037 (2)	0.079 (3)	−0.010 (3)	−0.028 (3)	0.002 (2)
C8	0.081 (3)	0.058 (3)	0.110 (4)	−0.032 (3)	−0.012 (3)	0.022 (3)
C5	0.078 (3)	0.046 (2)	0.056 (2)	0.0012 (19)	0.009 (2)	0.0072 (18)
C9	0.058 (2)	0.051 (2)	0.082 (3)	−0.0113 (18)	0.012 (2)	0.011 (2)
C6	0.125 (5)	0.052 (3)	0.054 (3)	0.017 (3)	−0.001 (3)	−0.002 (2)
O3	0.0519 (15)	0.0685 (17)	0.0521 (15)	−0.0016 (12)	0.0203 (12)	0.0067 (13)
O4	0.0683 (18)	0.105 (2)	0.0385 (15)	0.0053 (16)	0.0137 (13)	0.0178 (15)
O5	0.0421 (14)	0.0672 (17)	0.0668 (18)	−0.0069 (12)	0.0093 (12)	−0.0081 (14)
N2	0.0524 (17)	0.0380 (15)	0.0472 (17)	0.0036 (12)	0.0142 (14)	0.0113 (13)

### Geometric parameters (Å, °)

**Table:**

O1—C3	1.180 (4)	C2—H2B	0.9700
O2—C3	1.312 (4)	C7—C8	1.349 (8)
O2—H2	0.8200	C7—C6	1.352 (8)
N1—C1	1.492 (4)	C7—H7	0.9300
N1—H1A	0.8837	C8—C9	1.401 (7)
N1—H1B	0.8848	C8—H8	0.9300
N1—H1C	0.8849	C5—C6	1.396 (6)
C3—C2	1.512 (5)	C5—H5	0.9300
C4—C9	1.375 (5)	C9—H9	0.9300
C4—C5	1.378 (5)	C6—H6	0.9300
C4—C1	1.519 (5)	O3—N2	1.237 (4)
C1—C2	1.513 (5)	O4—N2	1.235 (4)
C1—H1	0.9800	O5—N2	1.260 (4)
C2—H2A	0.9700		
			
C3—O2—H2	109.5	C3—C2—H2B	109.3
C1—N1—H1A	109.0	C1—C2—H2B	109.3
C1—N1—H1B	109.4	H2A—C2—H2B	108.0
H1A—N1—H1B	109.4	C8—C7—C6	119.2 (4)
C1—N1—H1C	110.4	C8—C7—H7	120.4
H1A—N1—H1C	109.4	C6—C7—H7	120.4
H1B—N1—H1C	109.3	C7—C8—C9	121.7 (5)
O1—C3—O2	124.4 (3)	C7—C8—H8	119.2
O1—C3—C2	122.4 (3)	C9—C8—H8	119.2
O2—C3—C2	113.2 (3)	C4—C5—C6	120.0 (5)
C9—C4—C5	119.1 (4)	C4—C5—H5	120.0
C9—C4—C1	118.9 (4)	C6—C5—H5	120.0
C5—C4—C1	122.0 (3)	C4—C9—C8	119.2 (5)
N1—C1—C2	109.3 (3)	C4—C9—H9	120.4
N1—C1—C4	110.3 (3)	C8—C9—H9	120.4
C2—C1—C4	113.3 (3)	C7—C6—C5	121.0 (5)
N1—C1—H1	107.9	C7—C6—H6	119.5
C2—C1—H1	107.9	C5—C6—H6	119.5
C4—C1—H1	107.9	O4—N2—O3	120.2 (3)
C3—C2—C1	111.5 (3)	O4—N2—O5	119.3 (3)
C3—C2—H2A	109.3	O3—N2—O5	120.5 (3)
C1—C2—H2A	109.3		

### Hydrogen-bond geometry (Å, °)

**Table:**

*D*—H···*A*	*D*—H	H···*A*	*D*···*A*	*D*—H···*A*
C8—H8···O2^i^	0.93	2.56	3.414 (5)	152
C2—H2A···O4^ii^	0.97	2.46	3.256 (5)	140
O2—H2···O5^iii^	0.82	2.01	2.743 (4)	148
N1—H1C···O5^ii^	0.88	2.13	2.979 (4)	160
N1—H1C···O4^ii^	0.88	2.41	3.129 (4)	139
N1—H1B···O1^iv^	0.88	1.97	2.830 (4)	166
N1—H1A···O4	0.88	2.39	3.101 (4)	138
N1—H1A···O3	0.88	2.07	2.933 (4)	165

Symmetry codes: (i) −*x*+1, *y*−1/2, −*z*+1/2; (ii) *x*+1, *y*, *z*; (iii) *x*+1, −*y*+1/2, *z*+1/2; (iv) −*x*+2, *y*+1/2, −*z*+1/2.
